# Expression of the neuroprotective protein aryl hydrocarbon receptor nuclear translocator 2 correlates with neuronal stress and disability in models of multiple sclerosis

**DOI:** 10.1186/s12974-018-1290-6

**Published:** 2018-09-19

**Authors:** Tissa Rahim, Pierre Becquart, Maria-Elizabeth Baeva, Jacqueline Quandt

**Affiliations:** Department of Pathology and Laboratory Medicine, University of British Columbia, G227-2211 Wesbrook Mall, Vancouver, BC V6T 2B5 Canada

**Keywords:** Multiple sclerosis, Aryl hydrocarbon receptor nuclear translocator 2, Neuroprotection, Experimental autoimmune encephalomyelitis

## Abstract

**Background:**

Axonal degeneration and neuronal loss have been described as the major causes of irreversible clinical disability in multiple sclerosis (MS). The aryl-hydrocarbon receptor nuclear translocator 2 (ARNT2) protein has been associated with neuroprotection in models of ischemia and neuronal responses to stressors.

**Methods:**

To characterize its potential to influence inflammatory neurodegeneration, we examined ARNT2 expression in the experimental autoimmune encephalomyelitis (EAE) model of MS and characterized mediators that influence ARNT2 expression as well as plausible partners and targets.

**Results:**

*Arnt2* message and protein levels dropped significantly in EAE spinal cords as disease developed and were lowest at peak disability. ARNT2 expression is prominent in neuronal cell bodies within the gray matter with some staining in glial fibrillary acidic protein (GFAP)^+^ astrocytes in healthy animals. At peak disease, ARNT2 expression is reduced by 20–50% in gray matter neurons compared to healthy controls. ARNT2 intensity in neurons throughout the EAE spinal cord correlated inversely with the degree of immune cell infiltration (*r* = − 0.5085, *p* < 0.01) and axonal damage identified with SMI32 staining (*r* = − 0.376, *p* = 0.032). To understand the relationship between ARNT2 expression and neuronal health, we exposed enriched cortical cultures of neurons to hydrogen peroxide (H_2_O_2_) to mimic oxidative stress. H_2_O_2_ at lower doses rapidly increased ARNT2 protein levels which returned to baseline within 3–4 h. Exposure to higher doses of H_2_O_2_) dropped ARNT2 levels below baseline, preceding cytotoxicity measured by morphological changes and lactate dehydrogenase release from cells. Decreases in ARNT2 secondary to staurosporine and H_2_O_2_ preceded increases in cleaved caspase 3 and associated apoptosis. We also examined expression of neuronal pas 4 (*Npas4*), whose heterodimerization with ARNT2 drives expression of the neurotrophic factor brain-derived neurotrophic factor (*Bdnf*). Like ARNT2, *Npas4* levels also decline at the onset of EAE and are linked to decreases in *Bdnf*. In vitro, H_2_O_2_ exposure drives *Npas4* expression that is tied to increases in *Bdnf*.

**Conclusion:**

Our data support ARNT2 as a neuronal transcription factor whose sustained expression is linked to neuronal and axonal health, protection that may primarily be driven through its partnering with Npas4 to influence BDNF expression.

**Electronic supplementary material:**

The online version of this article (10.1186/s12974-018-1290-6) contains supplementary material, which is available to authorized users.

## Background

The aryl-hydrocarbon receptor nuclear translocator 2 (ARNT2) is a member of the basic-helix-loop-helix period-ARNT-single-minded protein (bHLH/PAS) transcription factor family [1]. ARNT2 heterodimerizes with other bHLH/PAS members to direct transcription of target genes in response to various environmental and physiological stimuli. These include hypoxia (hypoxia-inducible factor 1α (HIF-1α)), early cell determination/differentiation (single-minded homolog 1 (SIM1)) and environmental toxins (aryl hydrocarbon receptor (AhR)) [2]. Notably, AhR has been implicated in regulating pathogenic responses in an animal model of multiple sclerosis (experimental autoimmune encephalitis, EAE), mediating environmental influence on astrocytes and as a key component in the postulated mechanism of action for laquinimod (an MS disease-modulating therapy currently in clinical trials) [3–6].

ARNT2 is a nuclear transcription factor found almost exclusively in the central nervous system (CNS), but also localized to kidneys, the urinary tract, and the thymus [7–10]. ARNT2 was first described as an isoform of the aryl hydrocarbon receptor nuclear translocator (ARNT), named after its purported role in the nuclear translocation of the aryl hydrocarbon receptor (AhR). ARNT2 has been associated with neuroprotective properties in ischemic insults and cells undergoing oxidative damage. *Arnt2* gene transcript levels declined significantly following 2 h of recirculation post-ischemic injury, preceding neuronal death at 24 h of recirculation [10]. ARNT2 acts as an anti-apoptotic factor in PC12 cells (a cell line derived from a pheochromocytoma of the rat adrenal medulla), as downregulation of ARNT2 by RNA interference resulted in apoptosis of these cells, and oxidative stress-induced PC12 cell death was preceded by rapid and strong downregulation of ARNT2 [10]. *Arnt2* homozygous knockout mice and rats die perinatally (within the first day to 2 weeks after birth) secondary to a lack of hypothalamic neuroendocrine lineage formation, impaired regulation of HIF-1 target genes and thymic defects [11–14]. The importance of *Arnt2* has also been demonstrated in a case study of a family with a nonsense mutation in *Arnt2*, where children exhibited microcephaly and delayed or loss of myelination [15].

Physiological levels of synaptic activity result in a rapid and transient rise in calcium, which can activate a program of gene expression that promotes dendritic growth, synapse development, and neuronal plasticity, including but not limited to neuronal PAS domain protein 4 (NPAS4), a CNS-specific bHLH transcription factor that is the primary partner for ARNT2 in the CNS [16, 17]. Under excitatory conditions, a rapid and enhanced influx of calcium into the cell induces NPAS4 transcription, followed by heterodimerization with ARNT2, binding the PER-ARNT-SIM response element (PASRe) and guiding transcription of brain-derived neurotrophic factor (BDNF) at BDNF promoters I and IV of a total of nine promoters [18, 19]. This ARNT2:NPAS4 guidance of BDNF [20] transcription has been described under excitatory and neurodegenerative conditions [18, 19]. Notably, lack of NPAS4 and/or BDNF expression has been linked to neurodegenerative diseases and worsened disease stroke/ischemia models [21–23] and reductions of both *Npas4* and *Arnt2* mRNA have been observed in a rat model of depression [19].

Multiple sclerosis (MS) is a chronic inflammatory and demyelinating neurodegenerative disorder confined to the central nervous system (CNS) and characterized pathologically by alterations in the vasculature, inflammatory infiltrates, demyelination, glial scarring, oligodendrocyte loss, and axonal damage and loss [24]; the irreversible axonal loss and neurodegeneration is thought to be the major correlate of chronic disability in MS [25]. Recent investigations highlight that axonal injury starts early in the disease course [26, 27], and these pathological changes are confirmed by in vivo magnetic resonance imaging (MRI) studies showing early brain volume loss [28, 29].

Lymphocytes contribute to damage directly via cell-cell death or indirectly via soluble mediators including cytokines, antibodies, or proteases. The resident antigen-presenting cells, microglia, become activated, and recruited monocytes generate macrophages, the primary mediators of tissue injury and the most abundant inflammatory cells found in MS lesions [30, 31]. Oxidative stress, glutamate excitotoxicity, and inflammatory mediators, typically driven by surrounding glia/microglia, macrophages and lymphocytes, can all contribute to mitochondrial dysfunction [26, 32] and may contribute to disease progression [33, 34].

These disease mediators can induce rapid influx or release of calcium into the cell, which leads to activation of proteolytic enzymes, cleaved caspase processing initiating apoptosis, and damage to the mitochondrial membrane [35]. While many attribute demyelination and subsequent axonal loss as secondary to inflammatory cells and mediators in MS [26], this explains neither the neurodegeneration seen prior to inflammatory cell infiltration in animal models nor the progressive loss of function after the inflammatory phase has subsided [36, 37], or axonal loss despite intact myelin [37]. There is considerable interest in characterizing both gray matter disease and disease progression, where disability progresses despite suppression of new inflammatory activity [38]. Recent findings implicate mitochondrial dysfunction secondary to energy imbalance and increases in ROS as sources of oxidative damage not directly attributable to inflammation [39, 40] and suggest inflammation is secondary to more primary degenerative processes.

While the importance of ARNT2 in cell survival has been shown in models of ischemic injury, its expression patterns or role in chronic inflammatory and neurodegenerative disease of the CNS have not been characterized. We hypothesized that alterations in ARNT2 expression in vitro or in vivo in models of MS are associated with changes in cell viability and clinical disease, through processes relevant to pathogenesis in a model of MS. We report that *Arnt2* levels are significantly altered over the preclinical and acute phases of EAE compared to baseline and are lowest at times of peak disability. We found that ARNT2 expression in neuronal cells within the gray matter of EAE mice is lower at peak disease than observed in controls and propose that a loss in ARNT2 in EAE that is also tied to the loss of its primary CNS partner *Npas4* and downstream regulation of *Bdnf*, may be a primary driver for the neurodegeneration and disability seen in chronic inflammatory demyelinating disease. Decreases in neuronal ARNT2 correlate with the degree of inflammation and axonal damage. Furthermore, inflammatory and apoptotic mediators can influence neuronal expression of ARNT2 in vitro: ARNT2 protein increases in response to stressors, yet high doses or prolonged exposure is associated with decreases in ARNT2 that precede cell death and cleaved caspase 3 expression. This is the first report implicating ARNT2 in neurodegenerative processes relevant to inflammatory neurodegenerative disorders such as MS, and provides data to warrant its consideration as a primary contributor to disease development and progression.

## Methods

### EAE induction

All animal work was in compliance with our protocol approved by the University of British Columbia Animal Care Committee (certificate #A130281) and per the Canadian Council on Animal Care guidelines. Eight-week-old female C57BL/6 (Jackson Laboratories, Bar Harbor, ME) mice were immunized for chronic progressive EAE as previously described [41, 42] with 200 μg of MOG35-55 peptide (MEVGWYRSPFSRVVHLYRNGK; Stanford Pan Facility, Stanford, CA) in 4 mg/mL *Mycobacterium tuberculosis* (H37Ra) in incomplete Freund’s adjuvant (IFA) (Difco Laboratories, Detroit, MI). Pertussis toxin (200 ng, List Biologicals, Campell, CA) was delivered intraperitoneally that day and 2 days later. Mice were scored daily on a scale from 0 to 5 for severity of clinical symptoms: 0–0.5 indicated no disease/distal limp tail, 1.0 limp tail, 2.0 weakness in one or 2.5 in both hind limbs/slipping on bars, 3.0 paralysis in one or 3.5 both hind limbs, 4.0 hind limb paralysis plus weakness in one or in 4.5 both forelimbs, and 5 for moribund animals.

### Processing of EAE tissues

For qPCR and western blotting analyses, C57BL/6 mice spinal columns and brains were isolated from healthy mice and EAE mice immediately following CO_2_ exposure and decapitation at the following intervals: day 7, day 10 (pre-onset), day 14 (onset), day 18 (peak disease), day 25 (recovery), and days 32 and 45 which reflect some degree of worsening. Controls included healthy animals or sham/CFA-immunized (no MOG) animals that also received pertussis toxin harvested at days 7, 14, 25, and 45. For immunohistochemistry, animals were anesthetized with a ketamine/xylazine formulation, with subsequent transcardial perfusion with PBS followed by buffered formalin. Tissues were fixed for 3–5 days, and spinal cords removed before cryoprotection in 30% sucrose solution for at least 5 days. Spinal cords were cut into 5 mm pieces representing five to seven different levels and embedded in optimal cutting temperature compound (OCT) (VWR, Radnor, PA) and frozen until sectioning at 5 μM on a cryostat.

### Tissue staining and image acquisition

Sectioned tissues were permeabilized with 0.05% triton and blocked with 10% normal goat or donkey serum. Tissues were incubated with antibodies to NeuN, microtubule-associated protein 2 (MAP2), a neuronal marker) and glial fibrillary acidic protein (GFAP, a marker of astrocytes, all from EMD Millipore, Etobicoke, ON), myelin basic protein (MBP, Santa Cruz Biotechnology, Dallas, TX), SMI-32 (Biolegend, San Diego, CA) or ARNT2 (Santa Cruz) overnight. After incubation, tissues were washed with TBST followed by incubation with secondary antibody at room temperature. Normal goat, chicken, rat, mouse, and rabbit IgG (Santa Cruz) served as isotype controls.

Tissue sections and cells were visualized on a Zeiss Axio Vert 200 Inverted Fluorescence Microscope (Oberkochen, DE), and images acquired with an Axiocam 506 monochrome camera. Analysis was performed with Zeiss software Zen (Version 2.3). To quantify immunostaining results, sections from five to seven spinal levels spanning the entire cord (C4/5, T1/2, T5/6, T12/13, L5/6, S3/4) were examined from seven healthy/CFA control-immunized mice and five EAE mice at day 18 for a total of 26–40 sections per treatment group. Disease scores ranged from 2.5 to 4.0 with an average score of 3.25 ± 0.67, and all animals showed evidence of inflammatory infiltration of the spinal cord. Images were captured at × 20 with identical light intensity and exposure times applied to all images from each experimental set. ARNT2 expression in neurons was quantified by measuring the average intensity per pixel for ARNT2 over the area of NeuN-positive nuclei. First, a binary mask was created to select only the gray matter (based on regions of NeuN staining positivity) and then another one to select all nuclei of the section using the DAPI staining. Nuclei with a high fluorescence intensity (set at greater than 1500 mean intensity per pixel for NeuN) were selected, and the average fluorescence intensity per pixel for ARNT2 over the area of the DAPI-positive nucleus was measured. Neuronal cells were quantified by counting the NeuN/DAPI^+^ cells per square millimeter in the whole gray matter region, and ARNT2 intensity was quantified in cells in this larger region as well as those in the dorsal horn at each level based on anatomical outlines in the © 2008 Allen Institute for Brain Science Allen Spinal Cord Atlas available from http://mousespinal.brain-map.org. The values of all of the cells within one side of the spinal cord were first normalized to the average value for ARNT2 mean intensity measured in central canal ependymal cells (negative for ARNT2 expression) to account for any background staining between sections. The values for each spinal cord section within this region from each mouse were then pooled and averaged for analysis. The number of astrocytes in the gray matter was assessed using the same gray matter mask to select all the nuclei in the gray matter with DAPI staining. The nuclei with a high fluorescence intensity (greater than 1600 mean intensity per pixel) for GFAP were selected as GFAP^+^ cells and normalized to the surface area of the gray matter mask to obtain cell number per square millimeter.

### Assessment of inflammatory infiltrates, demyelination, and axonal damage

Immunostained sections from five levels of each EAE mouse spinal cord (*n* = 5, total of 25 levels) were assessed for the degree of inflammatory infiltration according to the criteria in Additional file 1: Table S1. MBP-negative regions of the white matter were outlined in Zen, and demyelinated areas (in μm^2^) were summed from each level and divided by the area of the entire white matter with the same binary mask approach used for gray matter to get a % area of demyelination for each level. SMI32-positive regions of interest, or spheroids, were selected as those regions with a staining intensity above the threshold set by averaging regions of white matter in healthy animals; the number of SMI32+ spheroids was evaluated per square millimeter of white matter at each of five levels from five EAE mice as for infiltrates. The relative sum of inflammation, % demyelination, and SMI32^+^ staining per level of the spinal cord was compared to the relative intensity of ARNT2 in neurons at that level.

### Primary cortical neuron-enriched cultures

Embryonic day 18 (E18) rat cortices were graciously provided by Dr. Shernaz Bamji (University of British Columbia) and were cultured as previously described [43]. Cortices were washed with 37 °C Hank’s Balanced Salt Solution (HBSS, Gibco, Carlsbad, CA), incubated in 0.25% Trypsin-EDTA (Sigma-Aldrich®, St. Louis, MO), DNase (Sigma-Aldrich) and HBSS, and dissociated and seeded at 50,000 cells/cm^2^ for western blotting/qPCR and 35,000 cells/cm^2^ for immunocytochemistry on poly-l-lysine-coated plates in Neurobasal® media (Thermo Fisher Scientific, Waltham, US) supplemented with B-27 (Gibco, Thermo Fisher Scientific). Cells were maintained at 5% CO_2_ and 37 °C until experiments at 7–16 days in vitro (DIV). Hydrogen peroxide (H_2_O_2_, Fisher, Waltham, MA) was used to mimic oxidative damage, and staurosporine (Sigma-Aldrich), a non-selective protein kinase inhibitor, was used to induce apoptosis, modeling pathological processes associated with degeneration in MS [44, 45].

### Cell lysis, protein quantification, and western blotting

Cells were lysed in radioimmunoprecipitation assay buffer (RIPA, tris-hydrochloric acid (MP Biomedicals, Santa Ana, CA), sodium chloride (Fisher), sodium deoxycholate (Fisher), NP-40 (Thermo Fisher Scientific, sodium dodecyl sulfate (SDS, MP Biomedicals), and EDTA (MP Biomedicals)) with 5% protease inhibitor (Roche, Basel, CH) and lysate stored at − 20 °C. Samples run on 12% tris/glycine gels (Bio-Rad, Hercules, USA) were transferred to nitrocellulose membranes, blocked with 2% skim milk, and incubated with primary antibodies overnight at 4 °C. Membranes were washed and incubated with secondary antibody at room temperature with Clarity™ Western ECL Blotting Substrate (Bio-Rad) added before imaging on a Bio-Rad ChemiDoc™ MP System. Blots were analyzed using quantitative densitometry on Image Lab™ (Bio-Rad 5.2) and normalized to tubulin as a loading control following normalization of data on different blots to a positive control spinal cord lysate that was loaded on each blot run; this enabled blot to blot comparisons when run in an identical manner and using the same exposure times.

### Reverse transcription and quantitative polymerase chain reaction

Tissues were processed with the AllPrep kit (Qiagen, Hilden, DE) per manufacturer guidelines and RNA stored until use. In vitro cell culture RNA was isolated using the Qiagen RNeasy Mini Kit, and protein was precipitated using buffer APP (Qiagen) per manufacturer’s guidelines). RNA was measured using a NanoDrop™ spectrophotometer (Wilmington, USA). Reverse transcription was performed using the QuantiTect® Reverse Transcription Kit (Qiagen) with Fast Start Essential DNA Green Master (Roche). cDNA, SYBR Green and ultrapure dH_2_O were combined and loaded on to LightCycler® 480 Multiwell plates (Roche) and run on a LightCycler® 480 Instrument II (Roche); qPCR primers are included in Additional file 1: Table S2. Data was subsequently analyzed using the LightCycler® 480 software (Version 1.5, Roche). Standard curves (*Arnt2*, *Npas4*, *Bdnf*, and *β-actin* cDNA generated from mouse brain, diluted in 10-fold successions) were included on each run for absolute quantification. Precipitated proteins from spinal cord were quantified and analyzed by western blotting as outlined above for cell cultures.

### Lactate dehydrogenase assay

Cell viability measurements were performed using a lactate dehydrogenase (LDH) cytotoxicity assay kit (Thermo Fisher Scientific™ Pierce™) per protocol guidelines. Controls included samples from dead cells (killed with lysis buffer provided for maximum LDH release) against which % cytotoxicity of treatments could be measured. Absorbance was measured using a Spectra Max plate reader (Molecular Devices, Sunnyvale, USA).

### Immunocytochemistry

Cells in culture were fixed with 10% phosphate-buffered formalin (Fisher), permeabilized with 0.1% Triton-X 100 (Fisher), and blocked with 10% normal goat serum (Gibco). Cells were washed with PBS, and incubated with primary antibodies for ARNT2, NeuN, MAP-2, GFAP, or cleaved caspase 3 (R&D Systems, Minneapolis, MN) in 2% normal goat serum overnight at 4 °C and secondary antibodies at RT (Additional file 1: Table S2). After counterstaining with 4′,6-diamidino-2-phenylindole (DAPI), images were acquired and analysis was performed with Zeiss software Zen (Version 2.3). ARNT2 staining was examined in NeuN-, GFAP-, or MAP-2-positive cells and cleaved caspase 3 in neuronal cells.

### Statistical analysis

All statistical analysis was performed using GraphPad Prism (version 5, La Jolla, CA). A repeated measures one-way ANOVA was performed, and if the data was not normally distributed, a repeated measures ANOVA on ranks (Friedman test) was performed when comparing multiple treatments. Both were followed with a Tukey multiple comparisons post-test. All data are reported as mean ± standard deviation unless otherwise indicated. For comparison of immunohistochemical evaluations in EAE to healthy/CFA-immunized mice, a Mann-Whitney comparison test was performed and significance reported at *p* < 0.05. The correlation of inflammation, MBP, and SMI32 staining to ARNT2 intensity was examined using a Pearson correlation.

## Results

### Arnt2 expression is altered over the course of EAE

**Fig. 1:**
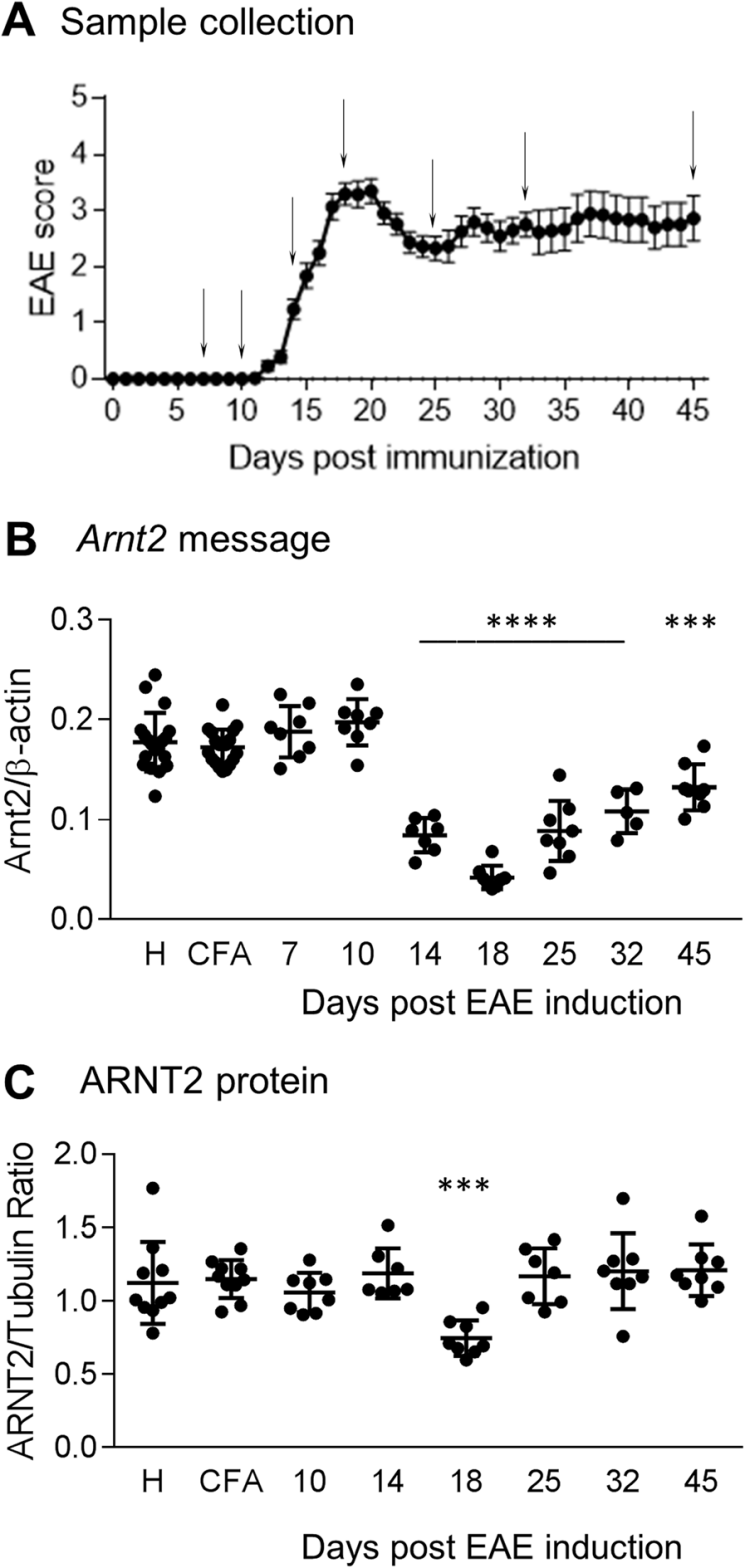
qPCR analyses of ARNT2 expression over the course of MOG-induced chronic EAE. **a** A cohort of 95 mice were induced for EAE or received only CFA and pertussis as sham-immunized healthy controls; healthy non-immunized mice also served as controls. Mice were scored as outlined in the Materials & Methods and points represent the mean score ± SEM of all mice with clinical symptoms. Tissues from 5 to 8 mice with similar disease scores were harvested at times indicated by arrows (days 7, 10, 14, 18, 25, 32, and 45) representing preclinical, onset, peak, recovery and times with some degree of worsening over time and analyzed by qPCR to obtain ARNT2 message levels normalized to β-actin (**b**) or analyzed by western blotting to quantify ARNT2 protein compared to tubulin (**c**). Each point represents the value obtained from one mouse; bars represent the mean ± SD. One way ANOVA followed by Dunnett’s multiple comparison test against CFA/sham immunized mice, ***p < 0.001, ****p < 0.0001

We began our investigation by characterizing *ARNT2* levels in the spinal cord where inflammation and degeneration are most prevalent in the MOG-induced model of MS. This is also the region where tissue changes are thought to contribute to disability in this model. qPCR analysis of chronic EAE spinal cord samples was performed with tissues from pre-onset, onset, peak, recovery/stabilization, and worsening stages of the disease compared to sham-immunized controls or healthy non-immunized littermates (Fig. 1a). Compared to CFA-immunized littermates, *Arnt2* levels in the spinal cord are decreased by onset (day 14) and are lowest at peak disease (Fig. 1b). Notably, *Arnt2* message levels have begun to increase again by day 25 where the animals have shown some recovery. By day 45 however, the RNA levels are still significantly below levels observed in healthy or sham-immunized animals. We also examined expression of the primary CNS partner for ARNT2, *Npas4*, and one of their downstream targets, *Bdnf*, over the course of EAE (Additional file 2). While *Arnt2* message tended to show increases prior to disease onset, both *Npas4* message and *Bdnf* message were elevated compared to sham-immunized animals prior to disease onset (at days 7 and 10 post EAE induction respectively). Like ARNT2, expression of *Npas4* and *Bdnf* decreased significantly over the course of EAE, with levels at their lowest at peak disability and recovering as animals recovered. Notably, ARNT2 protein was also lowest at peak clinical disease on day 18 (Fig. 1c) and was the only time point where ARNT2 protein levels were significantly different than healthy or sham CFA-immunized control mice.

### ARNT2 expression is altered during acute EAE

**Fig. 2:**
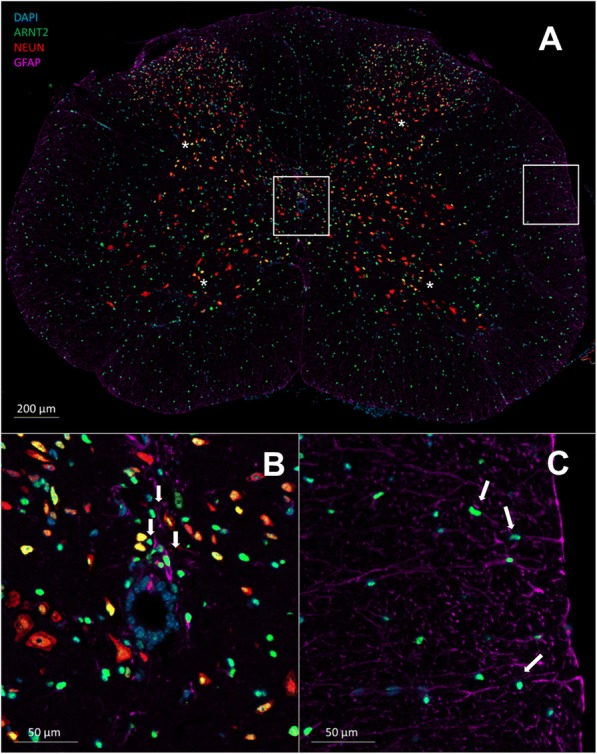
ARNT2 is localized to neurons and glia in the healthy mouse spinal cord. **a** A cross section of the mouse spinal cord at approximately C5 shows numerous ARNT2 (green) expressing cells throughout the gray and white matter of the cord. ARNT2 positivity colocalizing with NeuN^+^ (red) cells is observed frequently as yellow cells in the dorsal and ventral horns (asterisk) as well as throughout the intermediate zones. **b** An inset of the central canal from A shows ependymal cells of the central canal are negative for ARNT2, but indeed many GFAP^+^ cells in the area show positivity for ARNT2 (arrows). **c** In the white matter, ARNT2 positivity is often associated with GFAP^+^ astrocytes (arrows) with a fibrous phenotype in arrangements that extend away from the meninges

We next examined the expression of ARNT2 in the spinal cord of EAE animals at peak disease compared to controls, localizing ARNT2 to distinct cell populations and regions. In the spinal cords of sham-immunized animals, ARNT2 was localized to regions of both the gray as well as the myelin-rich white matter. In gray matter, ARNT2 staining was frequently associated with the nuclei of NeuN^+^ cells, either larger motor neurons in the ventral horns (arrows) or smaller cells of the dorsal horns or intermediate regions (arrowheads, Fig. 2a). While ependymal cells lining the central canal were negative for ARNT2 expression (Fig. 2b), ARNT2 was commonly associated with the nuclei of GFAP^+^ cells lining the midline and surrounding the central canal (insets, Fig. 2b, c respectively). ARNT2 positivity was also detected in several GFAP^+^ astrocytes lining the meninges.

**Fig. 3:**
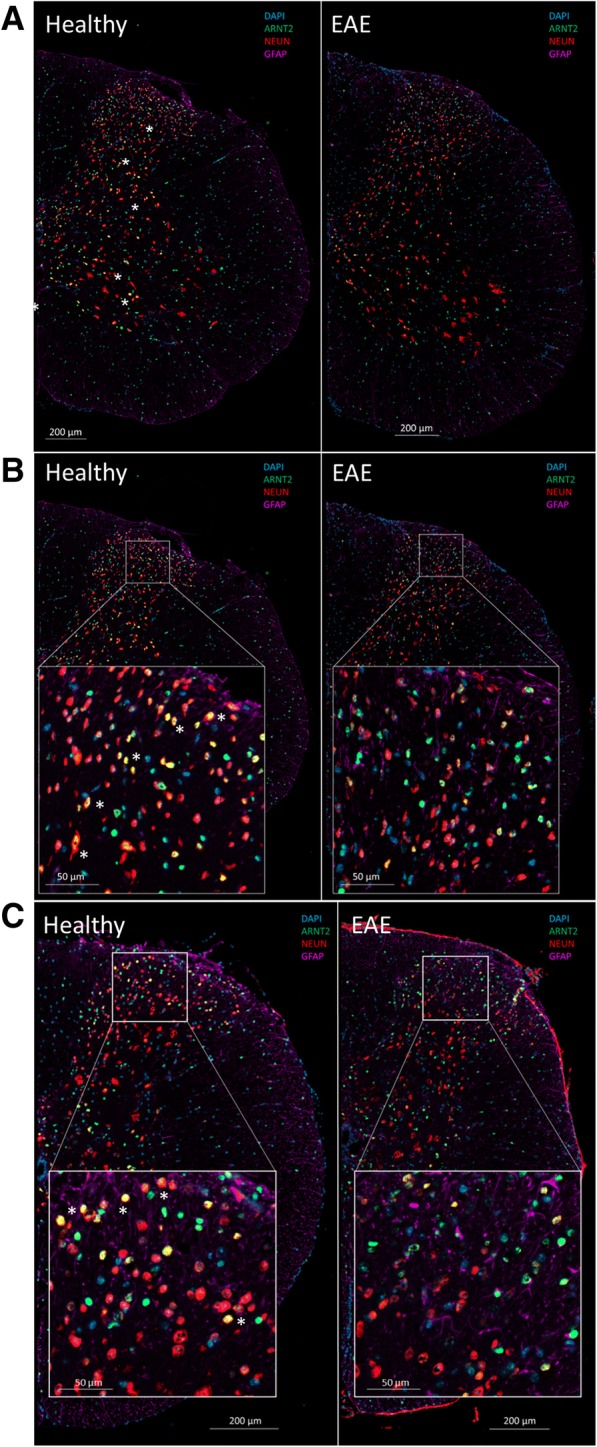
ARNT2 is expressed differentially between healthy immunized and EAE mice. Staining for ARNT2 in neuronal (NeuN^+^) or astrocytic (GFAP^+^) populations was localized predominantly to gray matter neurons and some astrocytes in the gray and white matter of healthy mice. **a** Compared to healthy mice, less NeuN+ cells stain for ARNT2 as well, giving rise to fewer yellow co-labeled cells (asterisk) in this C5 level image of EAE mice than observed in the healthy mice. With a focus on the dorsal horn (**b**), this loss in ARNT2 staining in NeuN^+^ cells seems even more pronounced; the same is true in **c** where images of the dorsal horn of EAE mice at T5/T6 show lower ARNT2 positivity in NeuN^+^ cells than observed in the dorsal horn of healthy mice (asterisk)

**Fig. 4:**
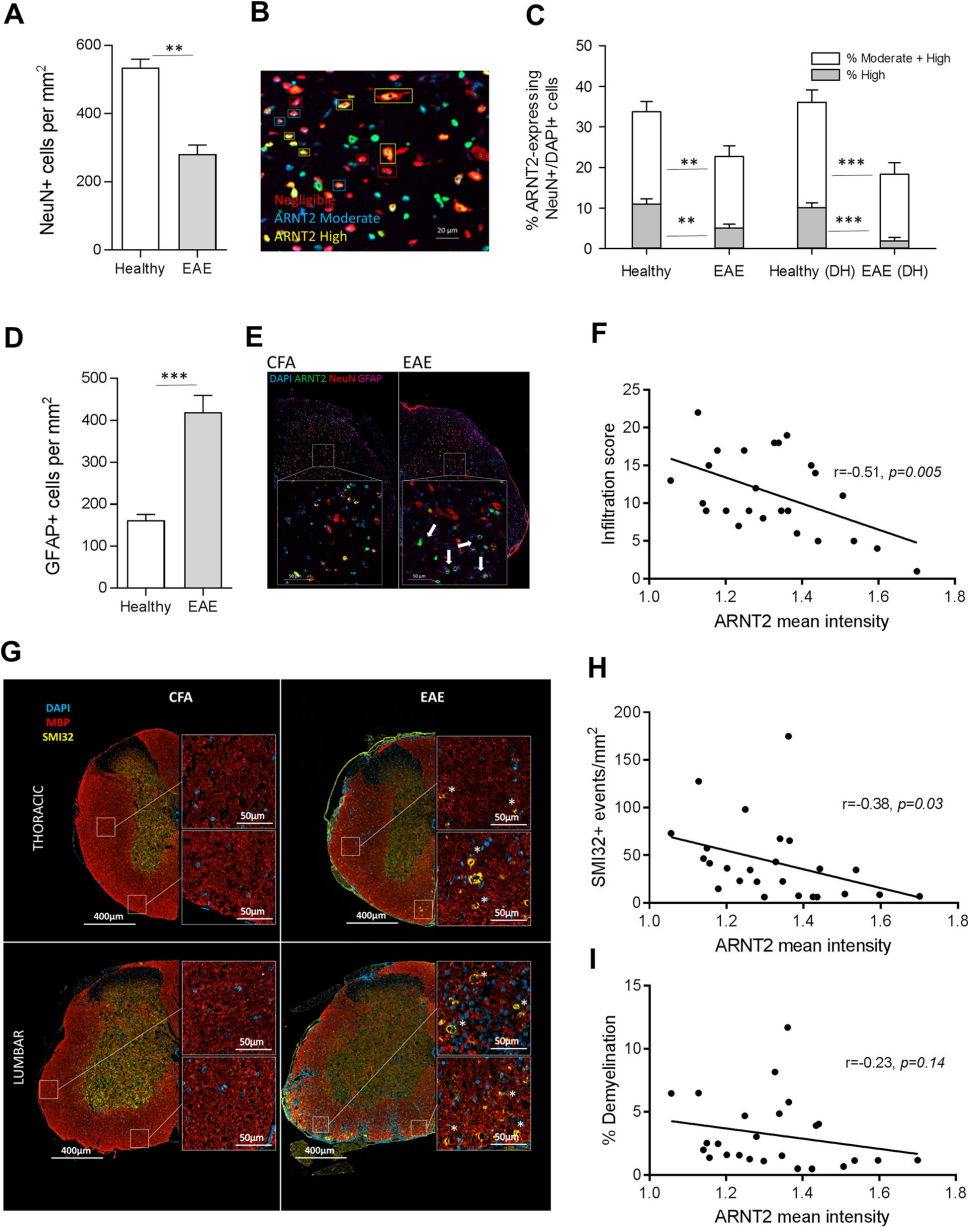
ARNT2 expression is decreased during peak disease in neuronal populations. **a** A relative loss of NeuN^+^ cells is observed in EAE mice compared to healthy sham-immunized controls examined in five to seven levels from three to four mice each. **b** Expression levels of ARNT2 were normalized to the staining of ependymal cells and categorized as high (mean intensities greater than 2× the background) or moderate (greater than 1.5× the background) as shown in representative images. **c** The % of NeuN^+^ cells expressing moderate or greater ARNT2 intensity was reduced by almost 30% in animals at peak disease. These differences were even greater in analyses of the dorsal horn (DH) regions in each section. Bars represent the mean intensity of 4–6 levels pooled from 7 healthy/CFA immunized animals (*n* = 39) compared to levels pooled from 5 EAE animals (*n* = 29) where whiskers indicate the SEM. *p* ≤ 0.05*, *p* ≤ 0.01**, *p* ≤ 0.001***, Mann-Whitney *T* test. **d** GFAP^+^ astrocytes were enumerated per mm^2^ of gray matter in each of the same levels analyzed in **c**. **e** A comparison of healthy to EAE animals at the L5/L6 level showed a marked increase in the frequency of GFAP^+^ cells in the gray matter of EAE mice; notably, these cells are largely positive for ARNT2 (arrows). **f** Each infiltrate was scored and pooled per each of 5 levels from 5 EAE mice. This infiltration score from 25 levels of EAE cord was compared to the mean intensity of ARNT2 in NeuN^+^ cells in the GM. **g** Serial sections from the same levels examined in C were stained for MBP and SMI-32. SMI32 positivity was not detected in sham-immunized animals, nor was a decrease in MBP staining. Associated with regions of infiltrates in EAE animals (inset), SMI32^+^ axons and spheroids were more frequent and in the lower areas of cord tended to be associated with regions of demyelination. In higher thoracic areas of the cord, SMI32 positivity was still present but demyelination was less prevalent. **h** The no. of SMI32+ events/mm2 and **i** % demyelination as calculated by masking regions devoid of MBP staining were correlated with ARNT2 mean intensity as outlined in **f**

Compared to the gray matter of healthy animals which typically showed numerous ARNT2^+^/NeuN^+^ cells, ARNT2 expression was detected at lower levels in the NeuN^+^ cells in EAE mice (Fig. 3). In the gray matter of an EAE spinal cord with moderate disability but at a higher level of the spinal cord (C4/5) with few infiltrates (Fig. 3a, b), there were far fewer double-staining NeuN^+^ cells in the EAE mouse than in the healthy mouse; this pattern was prominent in the ventral horn and intermediate zones of the gray matter though it seemed most pronounced in many levels within the dorsal horns (Fig. 3b, c insets). Notably, there was also a significant reduction in the number of NeuN^+^ cells/mm^2^ of gray matter in EAE animals compared to healthy controls (Fig. 4a). The reduction observed when samples were pooled from all levels of the cord (cervical, thoracic, and lumbar) was 535.9 ± 23.3 in healthy vs 281.7 ± 26.3 in EAE animals (*p* < 0.001); the greatest differences were observed in the thoracic and lumbar levels while differences in the cervical regions were not significant (430.7 ± 34.8 vs 389.9 ± 36.3, data not shown). Using a threshold for negative staining set by ARNT2 staining of the ependymal cells in each section, we measured the mean fluorescence intensity per pixel of each NeuN^+^ nucleus to compare healthy animals to EAE animals. We analyzed the entire gray matter and also performed a separate analysis of cells in the dorsal horn only. Separate from the determination of positive cells, we also distinguished the percent of moderate (> 1.5× negligible intensity threshold) versus highly staining cells (> 2× negligible intensity threshold) as illustrated in Fig. 4b. We did not detect a significant difference in the % of NeuN^+^ cells which were positive for ARNT2 between the two groups, 65.8 ± 3.1 versus 66.6 ± 3.4% in healthy vs EAE animals respectively over the entire gray matter region; numbers were also similar when only the dorsal horns were compared in the healthy (66.6 ± 3.4%) vs. EAE animals (63.0 ± 4.1%). However, the relative intensities of the stained cells were significantly lower in EAE animals than healthy mice (Fig. 4c). Over the entire gray matter region, the percentage of NeuN^+^ cells staining moderate to high was significantly lower in EAE animals (33.7 ± 2.6% of cells vs 22.6 ± 2.7%, *p* = 0.0065). This decrease was particularly marked in highly staining ARNT2 cells which dropped from 11.0 ± 1.4% of NeuN^+^ cells in healthy animals to 5.1 ± .9% of NeuN^+^ cells in EAE animals (*p* = 0.0024). Decreases in ARNT2 expression were even more pronounced within the dorsal horns; the proportion of moderate to highly stained NeuN^+^ cells dropped by 50% (36.0 ± 3.0% in healthy vs 18.2 ± 3.0% in EAE mice) whereas the proportion of highly expressing ARNT2 cells dropped by more than 80% (10.1 ± 1.7% in healthy vs 1.9 ± .9% in EAE mice). The number of GFAP^+^ cells in the gray matter of EAE mice was also significantly increased compared to healthy sham-immunized mice (Fig. 4d, e; 419.2 ± 40.4 vs 161.7 ± 14.1 cells/mm^2^, *p* < 0.0001). In Fig. 4f, notably, the intensity of ARNT2 expression in NeuN^+^ cells correlated inversely with the degree of immune cell infiltration. Declines in neuronal ARNT2 intensity were correlated with increased infiltrates (*r* = − 0.508, *R*^2^ = 0.259, *p* = 0.0047). In this EAE model, regions of immune cell infiltration often correlated with areas of demyelination (reduced staining for myelin basic protein, MBP) and also axonal damage which is apparent as axonal spheroids detectable by staining for non-phosphorylated neurofilaments with the SMI32 antibody [26, 46]. As expected, demyelination was not detected in our sham immunized animals (Fig. 4g). In contrast, all levels of EAE mice examined exhibited some degree of demyelination, typically associated with areas of inflammatory infiltration; the degree of demyelination ranged from 1.4 to 21.5% of the white matter area in a single level with an average of 4.7 ± 0.9% demyelination over all levels examined from EAE mice. Similarly, few SMI32^+^ axons were detected in sham-immunized animals at any level. In contrast, SMI32^+^ spheroids were detected in all levels of EAE animals examined, with an average of 62.7 ± 13.3 (range 6 to 300) spheroids per square millimeter. In EAE animals, we found a direct correlation between the frequency of SMI32^+^ spheroids and the degree of demyelination (*r* = 0.878, *R*^2^ = 0.772, *p* < 0.0001 (data not shown). Notably, the frequency of SMI32^+^ spheroids or axon “rings” stained with SMI32 (as in insets) per square millimeter correlated inversely with the ARNT2 intensity of NeuN^+^ cells at each level of the EAE mice examined (Fig. 4h; *r* = − 0.376, *R*^2^ = 0.141, *p* = 0.032). The degree of demyelination did not correlate with the ARNT2 intensity as closely throughout all levels examined (Fig. 4i; *r* = − 0.229, *R*^2^ = 0.052, *p* = 0.136). Examination of different levels of the spinal cord showed that SMI32^+^ were frequent in areas of extensive demyelination yet were also detected in areas where demyelination was less apparent, such as those in higher/thoracic levels of the cord (Fig. 4f, insets).

### Cell stressors alter ARNT2 protein levels

After establishing the relationship between inflammation and ARNT2 expression in vivo, we set out to examine factors which alter ARNT2 expression as well as the outcome of decreasing ARNT2 expression in vitro. Cultures routinely consist of 92.6 ± 6.9% MAP2^+^ neuronal cells (*n* = 3) by DIV10-14 (Additional file 3: Table S3) with some GFAP^+^ astrocytes (7.4 ± 6.9%). Healthy neurons, characterized by large cell bodies with complex axonal and dendritic processes, ranged in nuclear area from 50 to 200 μm^2^. Occasional astrocytes exhibited a reactive morphology with extensive ramifications in comparison to non-reactive astrocytes that are more flat and polygonal [47].

**Fig. 5:**
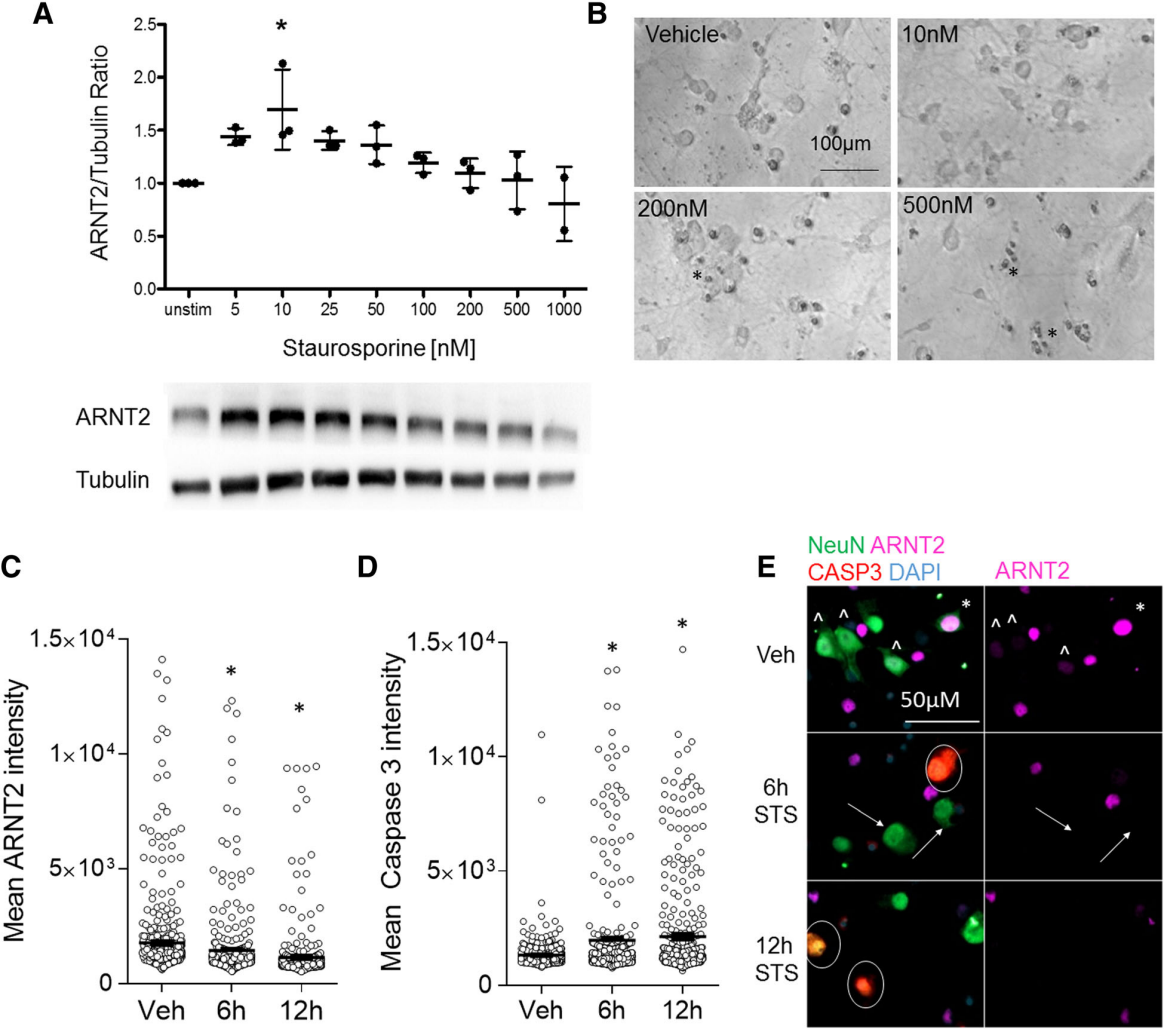
Staurosporine significantly increases ARNT2 protein levels. **a** Enriched neuronal cultures were treated with staurosporine at different doses for 24 h prior to analysis. Lines and whiskers indicate mean and standard deviation from results in three experiments with representative blots. *p ≤ 0.05 repeated measures ANOVA on ranks with Tukey’s multiple comparison test to vehicle (0.1% DMSO)-treated cells. **b** Phase microscopy images show dead/dying neurons following exposure to 500–1000 nM staurosporine. No change in % cytotoxicity as measured by LDH release was observed (data not shown). Enriched neuronal cortical cultures were treated with 1 μM staurosporine for 6 or 12 h and stained for NeuN to distinguish neurons for **c** ARNT2 and **d** cleaved caspase 3 intensity. Data points represent cells pooled from 11 random regions from wells treated in duplicate. *p ≤ 0.05 ANOVA followed by Bonferroni’s multiple comparisons test to vehicle-treated cells; bars represent the average mean intensity and whiskers the SEM. **e** Representative images from the wells analyzed in **c** and **d** reveal NeuN^+^ cells in vehicle-treated wells that stain brightly (asterisk) or faintly (circumflex accent) for ARNT2; cleaved caspase-3 staining (red) is negligible. With 6 or 12 h exposure to staurosporine (STS), ARNT2 intensity is significantly lowered in neurons (arrows) whereas Caspase3^+^ intensity increases in NeuN^+^ cells (circled)

Staurosporine potently induces apoptosis by preventing ATP binding to kinases, preventing protein kinase activity and resulting in growth factor withdrawal [48]. Reduced growth factor activity is linked to caspase-3 and cytochrome c activity, resulting in activation of the apoptotic pathway [49]. We tested staurosporine at a range of doses from 5 to 1000 nM for 24 h, where previous studies showed neuronal apoptosis in vitro with 500–1000 nM [48, 49]. Ten nanomolars of staurosporine increased ARNT2 levels by greater than 50% compared to vehicle-treated cells at 24 h (Fig. 5a, *n* = 3, *p* ≤ 0.05). At 500 to 1000 nM, cell death became evident by phase microscopy showing numerous cell bodies and cellular debris (Fig. 5b), which was not observed with ARNT2 levels nearer to vehicle controls. We next examined ARNT2 and cleaved caspase 3 intensities in enriched neuronal cultures and found that 6 or 12 h of exposure to 1 μM staurosporine significantly decreased ARNT2 expression and these decreases in ARNT2 were accompanied by increases in cleaved caspase 3 (Fig. 5c–e). In staurosporine-treated wells, cells staining brightly for cleaved caspase 3 typically showed negligible levels of ARNT2 compared to vehicle-treated controls.

### Oxidative stress significantly alters ARNT2 protein expression

**Fig. 6:**
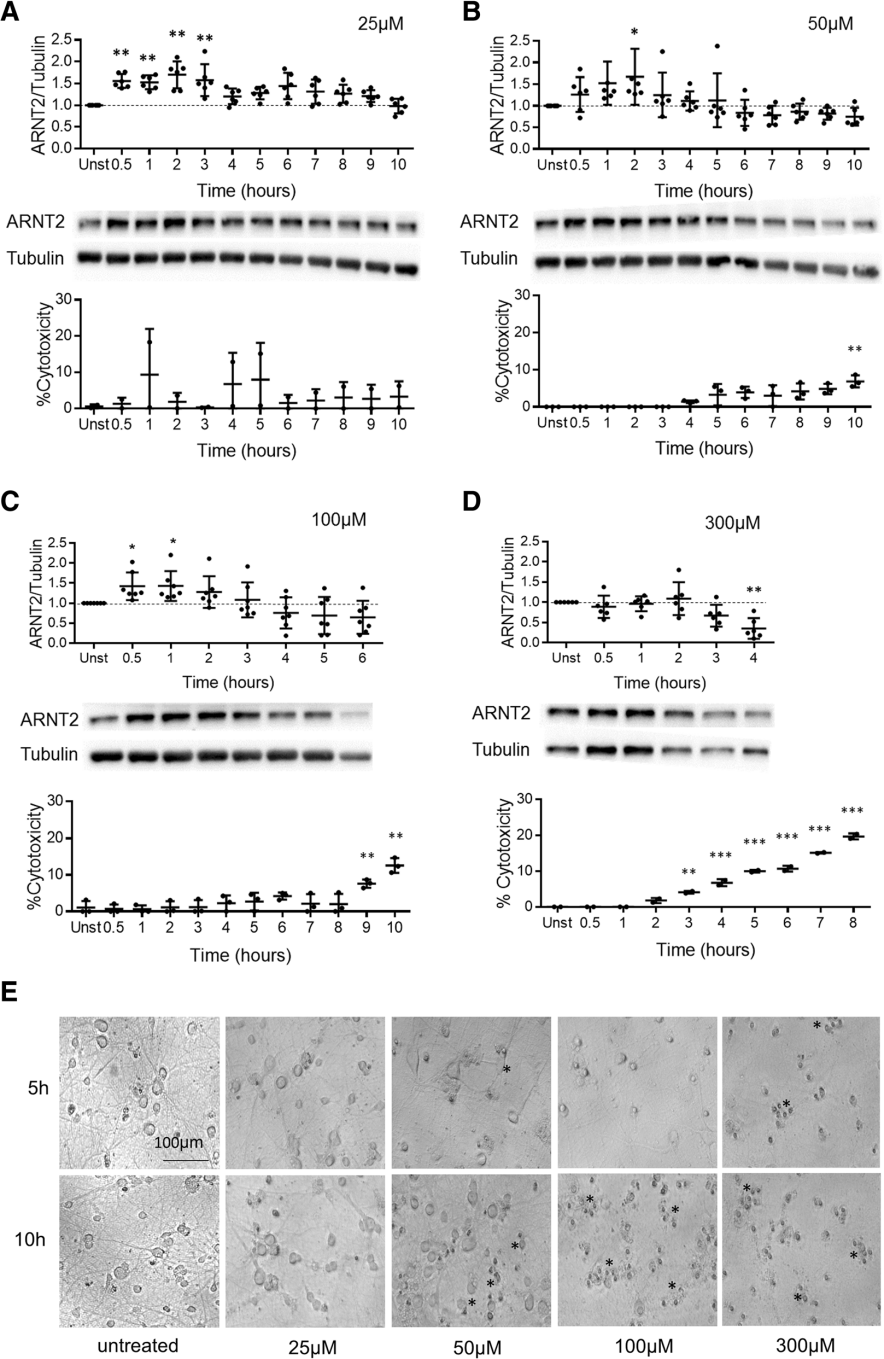
Hydrogen peroxide significantly alters ARNT2 protein expression. Four doses were applied: **a**–**d** 25, 50, 100, and 300 μM H_2_O_2_. Graphs represent the ARNT2/β-actin ratios of protein expression from western blotting. Points represent six biological replicates, with two technical replicates each, where lines and whiskers indicate mean and standard deviation, with representative blots. Cytotoxicity measured by lactic dehydrogenase (LDH) release is reported as the % cytotoxicity or % of total death relative to wells lysed with lysis buffer. Supernatants were collected at intervals following H_2_O_2_ treatment and compared to spontaneous LDH release in untreated controls. *p* ≤ 0.05*, *p* ≤ 0.01**, *p* ≤ 0.001***, repeated measures ANOVA on ranks with Tukey’s multiple comparison test to unstimulated. **e** Representative phase contrast photomicrographs show neuronal bodies with numerous complex dendrites and processes throughout the culture in untreated cultures. Addition of increasing doses of H_2_O_2_ over time leads to a loss in cell viability evidenced by decreasing processes and complexity of neurons with increasing amounts of cellular debris and condensed nuclei (asterisk)

To model mild to severe oxidative stress in primary cortical-enriched neuronal cultures, we applied 25, 50, 100, and 300 μM H_2_O_2_ (Fig. 6). Twenty-five micromolars of H_2_O_2_ was associated with a significant increase in ARNT2 tested over 0.5 to 3 h compared to untreated controls (Fig. 6a, *n* = 6, *p* ≤ 0.01). Notably, phase microscopy showed no changes in cell morphology or signs of blebbing and LDH release was also negligible over the 24 h treatment period (Fig. 6a). There were no changes in ARNT2 from 10 to 24 h in longer term experiments (data not shown).

Similar to 25 μM, 50 μM H_2_O_2_ increased ARNT2 protein levels with peak levels measured at 2 h after addition (Fig. 6b, *n* = 6, *p* ≤ 0.05); ARNT2 levels then returned to baseline by 3–4 h and ultimately dropped below baseline. The dropping of ARNT2 levels below baseline was not associated with morphological alterations within the first 5 to 10 h (Fig. 6e). Cell cytotoxicity measured by LDH release, while not apparent at 6 h, was significantly increased to 15–17% cytotoxicity by 12 and 18 h of cell exposure.

Exposure to a higher dose of H_2_O_2_ (100 μM) increased detectable ARNT2 protein at 0.5 and 1 h compared to controls (Fig. 6c, *n* = 7, *p* ≤ 0.05). By 3–4 h exposure, ARNT2 levels dropped below baseline in most experiments, but cells maintained their complex morphology through 5 h of treatment. Increases in LDH release became significant by 6 h (*p* ≤ 0.05) and were followed by a relative loss of dendrites and increased neuronal blebbing, nuclear condensation, and cell death apparent by 10 h (Fig. 6e). Notably, exposure to the highest tested dose H_2_O_2_ at 300 μM did not significantly increase ARNT2 levels and instead was associated with significant declines in ARNT2 expression beginning at 3–4 h which preceded blebbing of many cells by 5 h and loss of all dendritic morphology by 10 h (Fig. 6e).

We analyzed mRNA for *Arnt2*, *Npas4*, and *Bdnf* in H_2_O_2_-treated neuronal-enriched cultures compared to PBS-treated cells. Notably, *Arnt2* mRNA was not influenced by oxidative stress in primary cortical neuron-enriched cultures, indicating no relative change in transcript levels (Additional file 4). Over the time intervals where ARNT2 protein changed (0.5 to 2 h in response to H_2_O_2_), we saw no change in ARNT2 message. However, *Npas4* mRNA increased rapidly and specifically in response to oxidative stress and was followed closely by increases in *Bdnf* mRNA.

**Fig. 7:**
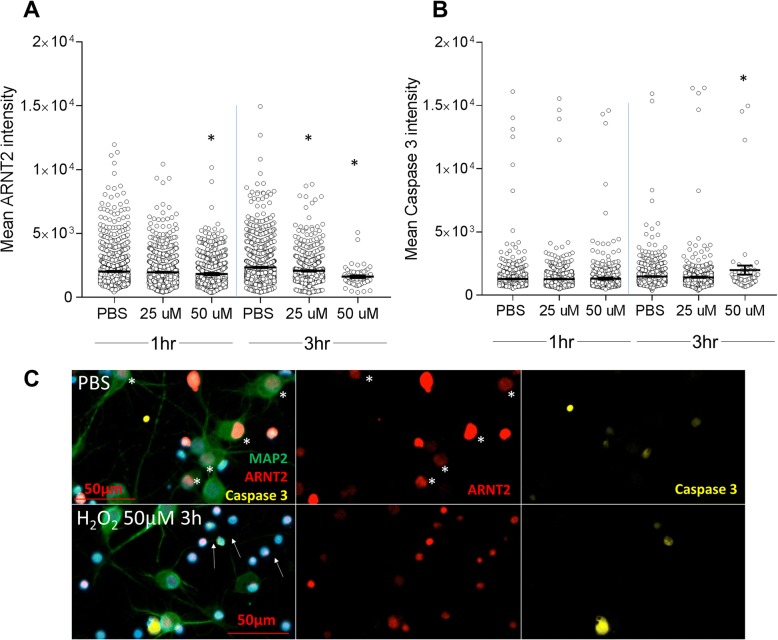
H_2_O_2_-treated neurons display decreased ARNT2 which precedes increases in cleaved-caspase 3 staining/apoptotic cell death. DIV7 neurons were treated with 25 or 50 μM H_2_O_2_ for 1 and 3 h and stained to examine ARNT2 and cleaved caspase-3 intensity. Data points represent cells pooled from 11 random regions from wells treated in duplicate. *p ≤ 0.05 ANOVA followed by Bonferroni’s multiple comparisons test to PBS-treated cells for the same treatment time; bars represent the average mean intensity and whiskers the SEM; 1 of 2 representative experiments with similar results is shown. **c** Representative images from **a** to **b** are shown. PBS-treated wells show numerous MAP2^+^ cells staining brightly or moderately for ARNT2 (asterisk); staining for cleaved caspase-3 is negligible. In contrast, exposure to 25uM H_2_O_2_ significantly reduced ARNT2, without associated increases in cleaved caspase-3 that are not detected until exposure to 50 μM H_2_O_2_ for 3 h. Fifty micromolars of H_2_O_2_ also increased the number of condensed nuclei and reductions in MAP2^+^ dendrites (arrow)

We next examined the relationship between declines in ARNT2 intensity and expression of cleaved caspase 3. Using early DIV7 neuronal cultures, we minimized the likelihood of glial contamination with the potential for MAP2 losses by neurons following exposure to stressors or undergoing cell death or apoptosis. Exposure to 25 μm H_2_O_2_ for 1 h had no effect on ARNT2 expression but 50 μM significantly reduced the mean ARNT2 intensity in neurons (Fig. 7a, *p* < 0.05). In this 1 h of exposure, there were no increases in cleaved caspase 3 expression in either group (Fig. 7b). In contrast, 3 h exposure to both 25 and 50 μM of H_2_O_2_ both significantly reduced ARNT2 intensity in neurons (Fig. 7c). Only exposure to the greater dose of H_2_O_2_ was associated with increases in cleaved caspase 3, although the majority of these cells also showed condensed nuclei (Fig. 7b, c) indicative of reduced cell viability that was also apparent in our earlier phase micrographs (Fig. 6e).

**Fig. 8:**
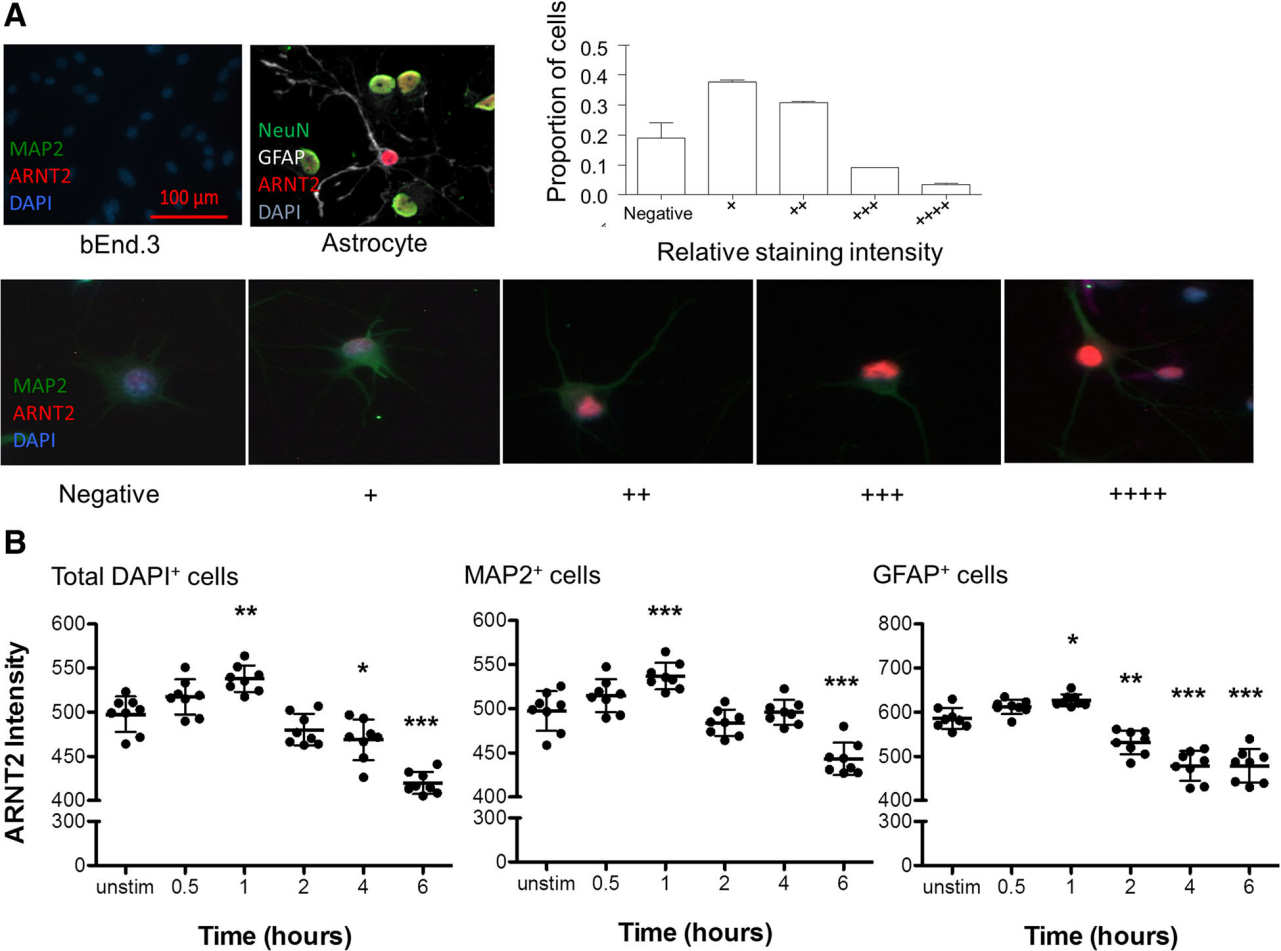
ARNT2 protein is localized to both neurons and astrocytes in neuronal-enriched cortical cultures. **a** Immunocytochemistry for ARNT2 reveals negligible ARNT2 staining in the Bend3 mouse immortalized brain microvascular endothelial cell line. Using threshold sets by the staining intensity in negative cells as described in the “Methods” section and Additional file 3, cells were divided according to staining intensity: negligible to ++++. The majority of MAP2^+^ neurons in the culture showed low to moderate staining for ARNT2; staining was also detected in the few contaminating GFAP^+^ astrocytes which demonstrated a reactive phenotype. One of three representative experiments is shown where eight regions from each of two duplicate wells were analyzed. **b** Following exposure to 100 μM H_2_O_2_, the entire enriched population as well as MAP2^+^ neurons and GFAP^+^ astrocytes examined individually demonstrated increases in ARNT2 staining intensity at 1 h; the same decreases after longer exposure observed with western blotting to baseline levels and below were replicated by immunocytochemistry. Points represent eight biological replicates, with two technical replicates each, where lines and whiskers indicate mean and standard deviation. *p* ≤ 0.05*, *p* ≤ 0.01**, *p* ≤ 0.001***, repeated measures ANOVA on ranks with Tukey’s multiple comparison test to unstimulated

We also examined ARNT2 expression at the individual cell level in our enriched neuronal cultures; 15–20% of the cells in the culture stained negligibly for ARNT2 (Fig. 8a). The majority of cells (60–70%) were low to moderate in their expression of ARNT2 while 10–15% had more intense ARNT2 staining. Notably, the few contaminating astrocytes were also positive for ARNT2. We tested the same doses of H_2_O_2_ (25 μM and 100 μM) previously used, focusing on earlier time points (0.5, 1, 2, 4, and 6 h). Total DAPI^+^ cells, MAP2^+^ cells, and GFAP^+^ cells were analyzed (Fig. 8b). With 100 μM H_2_O_2_, there was a significant increase in ARNT2 protein detected at 1 h exposure compared to untreated controls (*n* = 3, *p* ≤ 0.05 for DAPI^+^, 0.001 for MAP2^+^, and 0.01 for GFAP^+^ cells); this matched the increases in ARNT2 observed by western blotting (Fig. 6c). The staining intensity of ARNT2 was at or below baseline in neurons and astrocytes treated for 2–4 h and reached levels significantly lower than baseline (i.e., all three data groups, *n* = 3 for each; at 2 h: *p* ≤ 0.01 for GFAP^+^; at 4 h: *p* ≤ 0.05 for DAPI^+^, *p* ≤ 0.01 for GFAP^+^; at 6 h: *p* ≤ 0.001 for all three) which preceded changes in cell viability. Using this cell-specific analysis, we report a significant early upregulation of ARNT2 by 100 μM H_2_O_2_ in both neurons and astrocytes, which after extended exposure results in similar decreases in ARNT2 expression that precede cytotoxicity as in Fig. 6a–d.

## Discussion

ARNT2 has been characterized for its expression patterns and functional roles in vivo throughout development and under conditions of hypoxia, ischemic injury, and neuroprotection, as well as in in vitro models, including in primary cells and cell lines [10]. In this study, we set out to characterize changes in the amount and cellular localization of ARNT2 during inflammatory demyelinating disease of the CNS and to examine the ability of specific factors and stressors to alter ARNT2 protein expression in primary cortical neuron-enriched cultures.

We examined ARNT2 expression in both healthy and EAE animals in what is to our knowledge the first description of ARNT2 in the healthy spinal cord and in chronic inflammatory CNS disease. ARNT2 localization to NeuN^+^ neuronal cell bodies throughout gray matter in the healthy spinal cord was expected, with moderate to high expression observed throughout the gray matter in both the dorsal and ventral horns as well as in the intermediate zones. At peak disease, we observed a significant drop in ARNT2 expression in NeuN^+^ cells, associated with a loss of these cells throughout the gray matter. A loss of NeuN^+^ cells in the spinal cord during acute EAE has been described previously [50], but is not typically examined in most studies. Notably, decreases in ARNT2 expression correlated with increased numbers of inflammatory infiltrates. Given the regulation of ARNT2 in response to stressors and a role in neuroprotection, concurrent loss of ARNT2 expression and neuronal integrity and axonal damage evidenced by increased SMI32 positivity at peak disease highlights a potential role for ARNT2 in neuronal health and survival. In the chronic progressive model of EAE, we found a significant downregulation of *Arnt2* mRNA at peak disease in the spinal cord. Our histological data showed that loss of ARNT2 expression in spinal cord neuronal populations correlates with the drop in *Arnt2* message and protein that we observed in whole spinal cord lysates from preclinical to peak stages in our model.

Somewhat unexpectedly, we found ARNT2^+^/GFAP^+^ astrocytes lining the meninges, midline, and central canal of healthy animals with some scattering through the white matter. This is the first description of ARNT2 in astrocytes at brain interfaces in healthy animals, and increases in their number through the gray matter may implicate ARNT2^+^ astrocytes in processes mitigating damage with the potential to aid in repair that require further characterization. Astrocytes are a key source of neurotrophins throughout neuronal development and afford neuroprotection in response to trauma or insult; indeed, astrocytes express BDNF and this expression is upregulated under conditions of CNS injury [1]. ARNT2 expression by these cells in proximity to the meninges and proximal to the cerebrospinal fluid may define the ability to respond to stressors locally as well as those produced remotely. Expression of ARNT2 in astrocytes in vivo may not have been previously reported either because it was not detected in the specific brain regions examined, or a lower likelihood of detection may exist in healthy tissues. In our analyses, astrocytic ARNT2 expression in the white matter of the healthy spinal cord was relatively sparse and largely overshadowed by neuronal ARNT2 expression. During acute disease, ARNT2^+^ astrocytes morphologically resembled reactive astrocytes found in vivo in EAE as well as MS post-mortem tissue, with increased ramifications and high GFAP expression [51]. These cells are thought to play a role in the disease process through astrogliosis or expansion of astrocyte numbers due to nearby damage, causing scar formation and potential inhibition of axonal regeneration [51]. Preliminary stains to characterize GFAP^−^/ARNT2^+^ cells showed that ARNT2 was apparent in Olig2^+^ cells (a marker of oligodendrocytes but also present on reactive astrocytes [42] which warrants further investigation). In contrast, we could not detect ARNT2 in Iba-1+ microglia in healthy spinal cords nor in Iba-1+ cells in EAE spinal cords in preliminary analyses.

We report for the first time the effect of stressors involved in inflammatory and neurodegenerative processes on ARNT2 expression in primary enriched cultures of cortical neurons. We have shown that ARNT2 expression is increased under certain stress-initiating stimuli, including mild and more concentrated exposure to H_2_O_2_ and staurosporine. While ARNT2 protein is significantly increased by 25 to 100 μM H_2_O_2_ within a couple of hours, there is no change at the mRNA level in *Arnt2* over these doses or time points. This suggests early increases in ARNT2 protein are not secondary to enhanced mRNA transcription and subsequent translation. It may instead result from post-transcriptional/post-translational modifications or epigenetics, as described for other bHLH factors under developmental conditions [52, 53]. Indeed, epigenetic regulation of ARNT2 has previously been described in the context of neuronal differentiation of P19 cells, in which expression of ARNT2 was dependent upon downregulation of the expression of a repressor gene [2].

The upregulation of ARNT2 protein in primary cortical neurons in response to oxidative stress and apoptotic stimuli is a novel finding. Overall, ARNT2 protein levels in neurons appeared to follow two patterns when exposed to different levels of oxidative stress: an early upregulation with 25, 50, and 100 μM H_2_O_2_ and a return to constitutive levels versus exposure to higher doses (300 μM H_2_O_2_) where declining ARNT2 expression precedes losses in cell integrity as well as increases in cleaved caspase3 expression. A previous study has shown that pre-treatment of mouse hippocampal neurons with bicuculline reduced the percentage of cells undergoing apoptosis following staurosporine treatment which was abolished with knockdown of NPAS4 [54]. As the primary binding partner of NPAS4 in the CNS is ARNT2 and given the previous implication of ARNT2 as an anti-apoptotic factor in PC12 cells, it is plausible that their heterodimerization may play a role in protection of cells against apoptotic cell death [1, 10].

A neuroprotective role for ARNT2 is supported by both our in vitro and in vivo findings of changes in *Bdnf* that coincide with changes in *Npas4*. BDNF is a major mediator of physiological functions including activity-dependent functions, axonal growth, and a key molecule in neuronal and axonal survival with impact on memory, learning, and neurogenesis [55–59]. Npas4 controls activity-dependent Bdnf mRNA levels. BDNF expression is reduced by almost twofold in cultures expressing Npas4-RNA interference [17], and overexpressing dominant/negative forms of ARNT2 and Npas4 protein prevent BDNF induction [18]. BDNF has garnered particular interest in MS pathogenesis as one of only a handful of “protective” pathways described in MS models. In vitro, de novo expression of otherwise negligible *Npas4* in cortical neurons is driven rapidly by oxidative stress in our studies, a response which has not previously been described. This is closely associated with increases in *Bdnf* message, also an immediate early gene. Similarly, in vivo analyses of animals developing clinical disease secondary to autoimmune inflammatory neurodegeneration show that the presence of *Arnt2* RNA and protein prior to the onset of disease has the potential to partner with significant increases in *Npas4* RNA at day 7 to yield the observed increases in *Bdnf* RNA by day 10. As both ARNT2 message and protein decrease, similar decreases are seem with *Npas4* that are closely mirrored by reductions in *Bdnf*. Together, these findings demonstrate that the presence of ARNT2 can be closely linked to neurotrophic support via BDNF increases associated with the induction of Npas4.

## Conclusions

This study has uniquely characterized the expression of ARNT2 in an inflammatory neurodegenerative disease model of MS. We link changes in ARNT2 to inflammatory processes and mediators, where decreases are associated with losses in neuronal integrity in vitro and immune infiltration and axonal damage and disability in vivo. Our data support ARNT2 as a neuronal transcription factor whose sustained expression is linked to neuronal and axonal health, protection that may primarily be driven through its partnering with Npas4 to influence BDNF expression. This characterization of ARNT2 expression and regulation points to ARNT2 and its partner Npas4 as potential targets to effect neuroprotection and warrants further examination in inflammatory neurodegenerative disorders such as MS.

## Additional files

Additional file 1: **Table S1.** Scoring of inflammatory infiltrates. Each level of the spinal cord was examined for the number and nature of infiltrates; sections were the same as those used to assess ARNT2 expression. The total infiltrate score was determined by adding the lesion score for all lesions in that section to obtain a total infiltrate score for each level. **Table S2.** List of antibodies used for western blotting (WB), immunocyto- (IC) and immunohistochemistry (IH) and primers for qPCR. (DOCX 18 kb)

Additional file 2: Quantification of *Npas4* and *Bdnf* RNA in mouse spinal cords over the course of EAE. Tissues from 5 to 8 mice with similar disease scores were harvested at days 7, 10, 14, 18, 25, 32, and 45 as in Fig. 1a representing preclinical, onset, peak, recovery and times with some degree of worsening over time and analyzed by qPCR to obtain message levels normalized to β-actin. (PDF 52 kb)

Additional file 3: **Table S3.** Primary enriched neuronal cortical cultures have few glial cells. Breakdown of culture purity as well as cell distribution based on normalized ARNT2 intensity in primary cortical neuron-enriched cultures. Staining of enriched cortical cultures for neuronal (MAP2) and astrocytic (GFAP) markers. 3 independent experiments are shown to establish the threshold limits for ARNT2 staining intensity and distribution of staining intensities in MAP2^+^ cells in primary cortical neuron-enriched cultures. (DOCX 18 kb)

Additional file 4: qPCR to examine *Arnt2, Npas4, and Bdnf* expression following exposure of cortical neuronal cultures to H_2_O_2_. Values are normalized to β-actin. Bars represent average of 2 biological replicate/experiments. ARNT2 RNA levels remain largely unchanged. (PDF 295 kb)

## Abbreviations

ARNT2: Aryl hydrocarbon receptor nuclear translocator 2
BDNF: Brain-derived neurotrophic factor
bHLH/PAS: Basic-helix-loop-helix period-ARNT-single minded protein
Bic: Bicuculline
DAPI: 4′,6-Diamidino-2-phenylindole
EAE: Experimental autoimmune encephalomyelitis
GFAP: Glial fibrillary acidic protein
H_2_O_2_: Hydrogen peroxide
MAP2: Microtubule-associated protein 2
MOG: Myelin oligodendroglial protein
NPAS4: Neuronal pas domain protein 4
