# Supplementary material for: Expression of the neuroprotective protein aryl hydrocarbon receptor nuclear translocator 2 correlates with neuronal stress and disability in models of multiple sclerosis
Source: J Neuroinflammation. 2018 Sep 19;15:270. doi: 10.1186/s12974-018-1290-6 (PMC6145183; doi:10.1186/s12974-018-1290-6)
Supplement: Supplementary file 1 — Table S1. Scoring of inflammatory infiltrates. Each level of the spinal cord was examined for the number and nature of infiltrates; sections were the same as those used to assess ARNT2 expression. The total infiltrate score was determined by adding the lesion score for all lesions in that section to obtain a total infiltrate score for each level. Table S2. List of antibodies used for western blotting (WB), immunocyto- (IC) and immunohistochemistry (IH) and primers for qPCR. (DOCX 18 kb) [file 12974_2018_1290_MOESM1_ESM.docx]

|  | No infiltrates | Mild  <25 infiltrated cells | Moderate  25-50 infiltrated cells | Large  50-100 infiltrated cells | Severe/extensive parenchymal  100+ cells |
| --- | --- | --- | --- | --- | --- |
| **Lesion score** | 0 | 1 | 2 | 3 | 4 |

**Additional file 1: Table S1** Scoring of inflammatory infiltrates. Each level of the spinal cord was examined for the number and nature of infiltrates; sections were the same as those used to assess ARNT2 expression. The total infiltrate score was determined by adding the lesion score for all lesions in that section to obtain a total infiltrate score for each level.

| Primary Antibody | Species | Catalog Number | Dilution | Use |
| --- | --- | --- | --- | --- |
| ARNT2 | Rabbit | sc-5581 | 1:250 (IC/IH)  1:2000 (WB) | WB/IC/IH |
| GFAP | Chicken | AB5541 | 1:2500 (IC)  1:2000 (IH) | IC/IH |
| MAP2 | Mouse | M9942 | 1:750 | IC/IH |
| NeuN | Mouse | MAB377 | 1:2700 | IH |
| Tubulin | Rat | MAB1864 | 1:1000 | WB |
|  |  |  |  |  |
| SMI-32 | Mouse | 801701 | 1:1000 | IH |
|  |  |  |  |  |
| MBP | Goat | SC-13914 | 1:400 | IH |
|  |  |  |  |  |
| **Secondary Antibody** | **Catalog Number** | **Dilution** | **Wavelength (nm)** | **Use** |
| Goat anti-rabbit | A11036 | 1:500 | 568 | IC/IH |
| Goat anti-chicken | A11036 | 1:1000 (IC)  1:500 (IH) | 647 | IC/IH |
| Goat anti-mouse | A11029 | 1:500 | 488 | IC/IH |
| Goat anti-rabbit | 111-035-045 | 1:2000 | N/A | WB |
| Donkey anti-rat | 712-035-153 | 1:2000 | N/A | WB |
| Donkey anti-goat | A11057 | 1:500 | 568 | IH |
| Donkey anti-mouse | A31571 | 1:500 | 647 | IH |
| Donkey anti-rabbit | A21206 | 1:500 | 488 | IH |
| **Normal IgG Species** | **Catalog Number** | **Dilution** |  | |
| Rabbit | Sc-5581 | 1:500 |  |  |
| Chicken | AB5541 | 1:1000 |  |  |
| Mouse | M9942 | 1:160 |  |  |
| Goat | AB-108-C | 1:4000 |  | |

| **Gene** | **Forward (5’ – 3’)** | **Reverse (5’ – 3’)** |
| --- | --- | --- |
| NPAS4 | CTC TTC CTG GCC ATG TTC CAG AGC | TCA GCC AAC AGG CGG TAG TGT T |
| ARNT2 | CCA GTC TTG CCA ACA GGA CTC C | AGC ATG TCC TGG AAC ACT TCA GTC |
| BDNF | AGT CAA GTG CCT TTG GAG CCT | TAC TGT CAC ACA CGC TCA GCT |
| ACTB | CCA GCC TTC CTT CTT GGG TAT | TGT GTT GGC ATA GAG GTC TTT ACG |

**Additional file 1: Table S2** List of antibodies used for western blotting (WB), immunocyto- (IC) and immunohistochemistry (IH) and primers for qPCR.
