# Supplementary figures and images for: Expression of the neuroprotective protein aryl hydrocarbon receptor nuclear translocator 2 correlates with neuronal stress and disability in models of multiple sclerosis
Source: J Neuroinflammation. 2018 Sep 19;15:270. doi: 10.1186/s12974-018-1290-6 (PMC6145183; doi:10.1186/s12974-018-1290-6)

**A** *Npas4* message

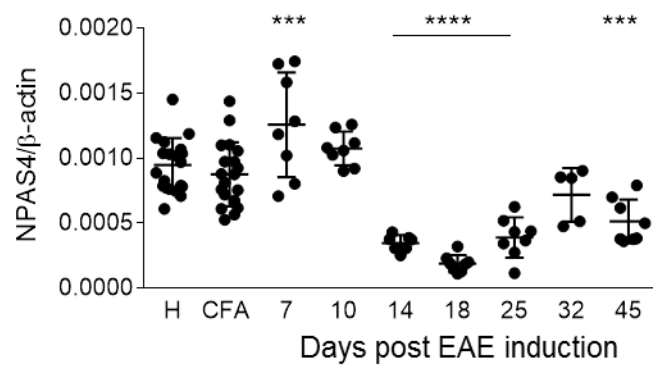

**B** *Bdnf* message

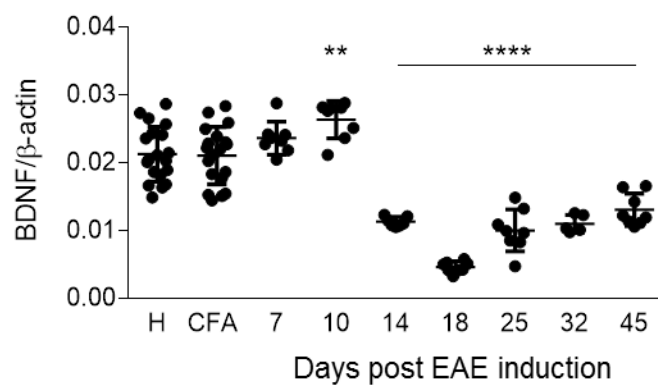

Supplement: Supplementary file 2 — Quantification of Npas4 and Bdnf RNA in mouse spinal cords over the course of EAE. Tissues from 5 to 8 mice with similar disease scores were harvested at days 7, 10, 14, 18, 25, 32, and 45 as in Fig. 1a representing preclinical, onset, peak, recovery and times with some degree of worsening over time and analyzed by qPCR to obtain message levels normalized to β-actin. (PDF 52 kb) [file 12974_2018_1290_MOESM2_ESM.pdf]

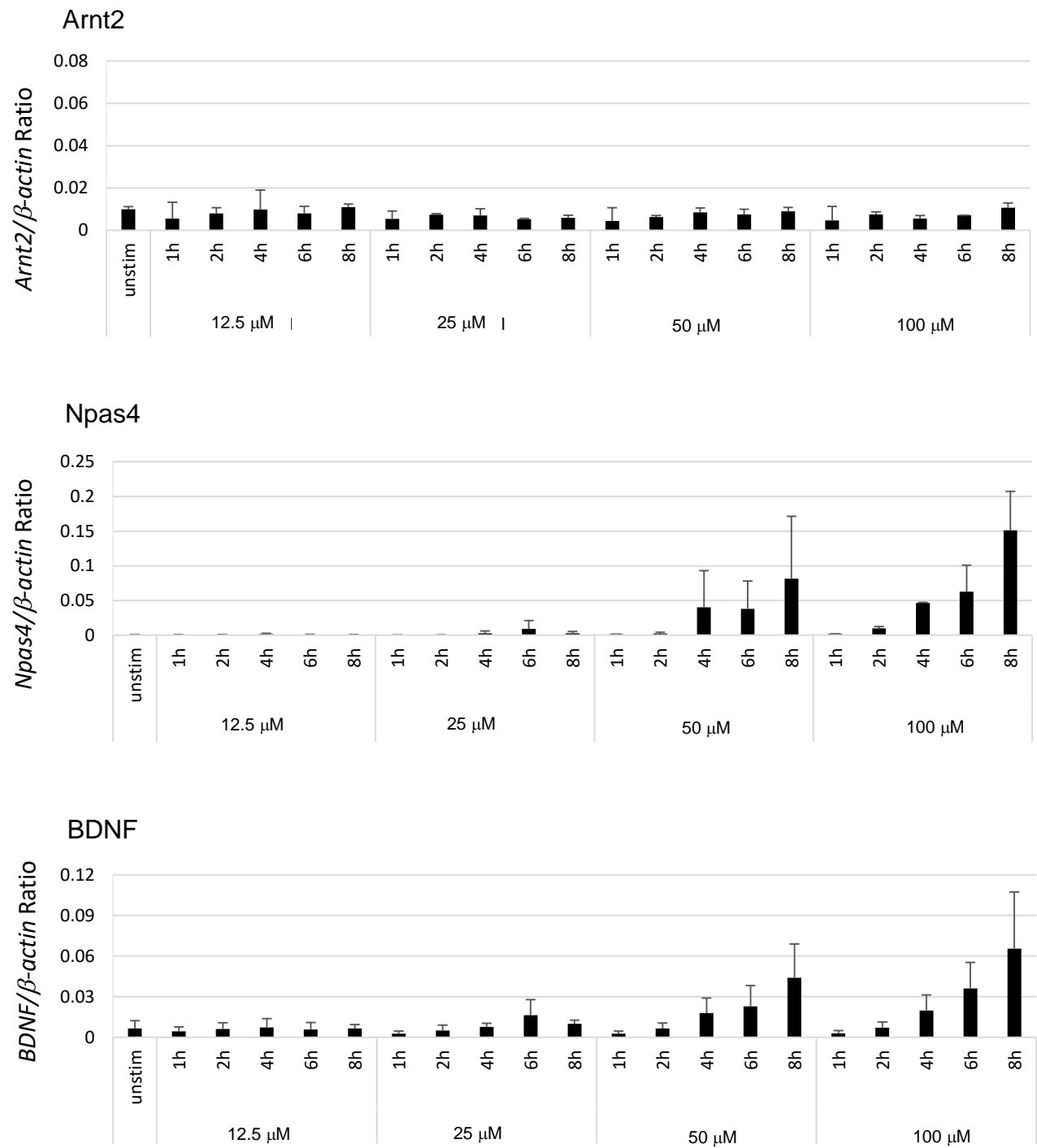

Supplement: Supplementary file 4 — qPCR to examine Arnt2, Npas4, and Bdnf expression following exposure of cortical neuronal cultures to H2O2. Values are normalized to β-actin. Bars represent average of 2 biological replicate/experiments. ARNT2 RNA levels remain largely unchanged. (PDF 295 kb) [file 12974_2018_1290_MOESM4_ESM.pdf]
