# Supplementary material for: Expression of the neuroprotective protein aryl hydrocarbon receptor nuclear translocator 2 correlates with neuronal stress and disability in models of multiple sclerosis
Source: J Neuroinflammation. 2018 Sep 19;15:270. doi: 10.1186/s12974-018-1290-6 (PMC6145183; doi:10.1186/s12974-018-1290-6)
Supplement: Supplementary file 3 — Table S3. Primary enriched neuronal cortical cultures have few glial cells. Breakdown of culture purity as well as cell distribution based on normalized ARNT2 intensity in primary cortical neuron-enriched cultures. Staining of enriched cortical cultures for neuronal (MAP2) and astrocytic (GFAP) markers. 3 independent experiments are shown to establish the threshold limits for ARNT2 staining intensity and distribution of staining intensities in MAP2+ cells in primary cortical neuron-enriched cultures. (DOCX 18 kb) [file 12974_2018_1290_MOESM3_ESM.docx]

|  | %  neurons  (mean ± std) | % astrocytes (mean ± std) | Isotype control  (mean pixel/total pixel count ± std ) | Positivity cut-off  (mean pixel/total pixel count) | + | ++ | +++ | ++++ |
| --- | --- | --- | --- | --- | --- | --- | --- | --- |
| **1** | 85.3±5.1 | 14.6±4.7 | 287.2±24.3 | 335.8 | 335.8-500 | 500 - 600 | 600 -  700 | 700+ |
| **2** | 93.4±0.2 | 6.5±1.0 | 372.4±7.549 | 387.5 | 387.5-500 |  |  |  |
| **3** | 89.4±0.02 | 10.5±1.7 | 355.0±15.4 | 385.8 | 385.8-500 |  |  |  |

**Additional file 3: Table S3** **Primary enriched neuronal cortical cultures have few glial cells.**  Breakdown of culture purity as well as cell distribution based on normalized ARNT2 intensity in primary cortical neuron-enriched cultures. Staining of enriched cortical cultures for neuronal (MAP2) and astrocytic (GFAP) markers. 3 independent experiments are shown to establish the threshold limits for ARNT2 staining intensity and distribution of staining intensities in MAP2^+^ cells in primary cortical neuron-enriched cultures.
